# The formation of a hatching line in the serosal cuticle confers multifaceted adaptive functions on the eggshell of a cicada

**DOI:** 10.1186/s40851-021-00178-8

**Published:** 2021-05-13

**Authors:** Minoru Moriyama, Kouji Yasuyama, Hideharu Numata

**Affiliations:** 1 National Institute of Advanced Industrial Science and Technology (AIST), Tsukuba, 305-8566 Japan; 2 Graduate School of Science, Osaka City University, Osaka, 558-8585 Japan; 3 Kawasaki Medical School, Kurashiki, 701-0192 Japan; 4 Kawasaki University of Medical Welfare, Kurashiki, 701-1093 Japan; 5 Graduate School of Science, Kyoto University, Kyoto, 606-8502 Japan

**Keywords:** Chorion, *Cryptotympana facialis*, Desiccation tolerance, Embryonic diapause, Hatching line, Serosal cuticle, Water loss

## Abstract

Insect eggshells must meet various demands of developing embryos. These demands sometimes conflict with each other; therefore, there are tradeoffs between eggshell properties, such as robustness and permeability. To meet these conflicting demands, particular eggshell structures have evolved in diverse insect species. Here, we report a rare eggshell structure found in the eggshell of a cicada, *Cryptotympana facialis*. This species has a prolonged egg period with embryonic diapause and a trait of humidity-inducible hatching, which would impose severe demands on the eggshell. We found that in eggs of this species, unlike many other insect eggs, a dedicated cleavage site, known as a hatching line, was formed not in the chorion but in the serosal cuticle. The hatching line was composed of a fine furrow accompanied by ridges on both sides. This furrow-ridge structure formed in the terminal phase of embryogenesis through the partial degradation of an initially thick and nearly flat cuticle layer. We showed that the permeability of the eggshell was low in the diapause stage, when the cuticle was thick, and increased with degradation of the serosal cuticle. We also demonstrated that the force required to cleave the eggshell was reduced after the formation of the hatching line. These results suggest that the establishment of the hatching line on the serosal cuticle enables flexible modification of eggshell properties during embryogenesis, and we predict that it is an adaptation to maximize the protective role of the shell during the long egg period while reducing the barrier to emerging nymphs at the time of hatching.

**Supplementary Information:**

The online version contains supplementary material available at 10.1186/s40851-021-00178-8.

## Background

The development of the eggshell, a multilayered envelope composed mainly of chorion, is considered indispensable for insect expansion and diversification in terrestrial habitats [1–3]. As an interface between postzygotic cells and external environments, eggshells must face various challenges that potentially conflict with each other. Because of their immobility, small size, and high surface-to-volume ratio, insect eggs are usually at risk for a variety of environmental stresses such as physical damage, predation, desiccation, pathogen intrusion, and drowning. In this regard, physically robust and chemically impermeable eggshells appear to be favored to maximize their role as a barrier. At the same time, it is necessary for eggshells to be permeable so that embryos can access external resources through. The exchange of oxygen and carbon dioxide is essential for aerobic respiration, but the ability of eggshells to perform it becomes a cause of water loss because of the small molecular size of water [4, 5]. Many insects need to absorb environmental water in a liquid or vaporous state during embryogenesis [6–9], and in some species, nutrients may be absorbed from the surrounding substrate [10, 11]. In addition, robust eggshells can be a physical obstacle when neonates emerge from them [12, 13]. These demands favor permeable and fragile eggshells, although such properties necessarily impair the protective functions of the shell. Therefore, tradeoffs between these properties have evolved and diversified under species-specific selection pressure [14, 15] and have been fine-tuned along with specific life cycle strategies even within individual species [16–18]. In addition, innovative adaptations have evolved in insect eggshells to make these conflicting demands compatible. For example, the chorion layer often possesses aeropyles or air cavities, which restrict the surface area to minimize water loss while maximizing gas exchange capacity [1, 2]. Chorionic extensions, such as respiratory horns and appendages, reinforce gas exchange, especially in air-limited environments [1, 19]. To facilitate eggshell rupture at hatching, eggs of many species possess a special region of weakness on the chorion, called the operculum or hatching line [2, 20–22]. During embryogenesis, the serosal cuticle can be positioned beneath the chorion layer and augment or replace functions of the eggshell [23, 24]. This additional membrane can confer superior desiccation tolerance [25–27]. In some species, the specialized serosa and serosal cuticle, called the hydropyle, offer a route for water absorption [28–30].

**Fig. 1:**
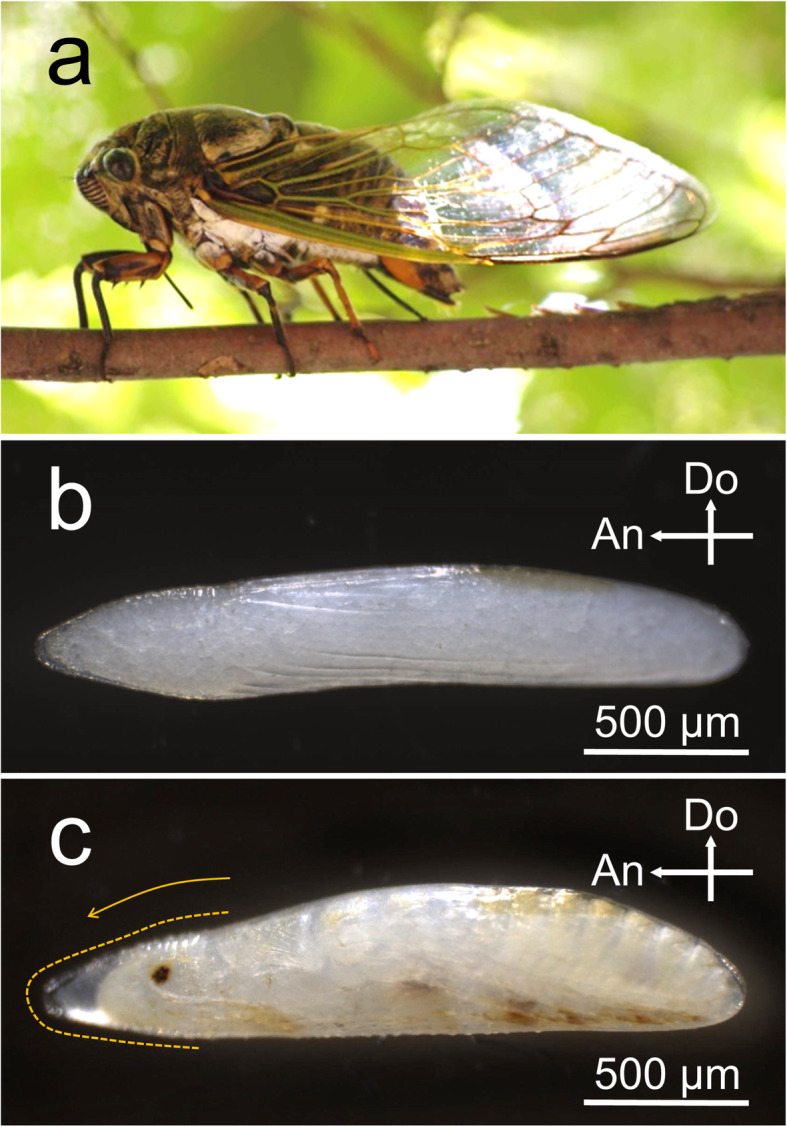
Eggs of *Cryptotympana facialis*. **a** An adult female of *C. facialis* laying eggs in a dead twig. **b** An egg on the day of oviposition. **c** An egg with a fully developed embryo. An orange broken line indicates the site of eggshell cleavage (hatching line), and an orange arrow indicates the cleavage direction. Crossed arrows indicate the anterior (An) and dorsal (Do) directions

In this study, we present a unique feature that is found in eggshells of cicadas and enables these insects to meet the conflicting demands of their embryonic lives. Cicada eggs are laid into a small hole made in plant tissues by a spear-shaped ovipositor [31–33]. A remarkable feature of the eggs of temperate cicadas is the extremely long duration of the egg stage; in some species, the egg period reaches up to 12 months [31, 34, 35]. Thus, the eggs are expected to face a sequence of environmental stresses over the course of the seasons. Another unique trait of cicada eggs is that even after the completion of embryogenesis, nymphs remain in the eggshell, rapidly emerging from it in response to high humidity cues derived from rain [36, 37]. We speculated that such unique life cycle traits impose severe demands for robustness and permeability on cicada eggshells and can be associated with a functional specialization of eggshell structures to meet these demands. In this study, we first examined the ultrastructure of the eggshell during the course of the prolonged egg period in *Cryptotympana facialis* (Fig. 1a), whose embryonic development and hatching traits have previously been investigated [34–38]. Then, we found a peculiar structure rarely seen in insect eggs: a structure resembling a hatching line was built into the serosal cuticle. We further investigated the morphogenesis of this structure along with accompanying changes in eggshell properties. The findings suggested that this peculiar structure contributes to flexible changes in eggshell properties that aid in fulfilling various conflicting demands faced by cicada eggs.

## Methods

### Collection and maintenance of cicada eggs

Adult females of *C. facialis* were collected on the campus of Osaka City University, Osaka, Japan. *Cryptotympana facialis* normally lays eggs in dead twigs of woody plants (Fig. 1a) [33]. In order to obtain loose eggs, the collected adult females were wrapped in a wet paper towel as described previously [34]. Eggs laid in the folds of the paper towel were picked out every day and put in a plastic Petri dish (6 cm diameter and 1 cm depth). They were kept in a sealed plastic container (13 cm diameter and 9 cm depth) humidified with pieces of wet cotton and maintained at 25 °C under a photoperiod of 16 h of light and 8 h of dark (16L8D). After a 60-day incubation under these conditions, all eggs reached the diapause stage, and embryogenesis stopped [34]. Then, embryonic diapause was terminated by keeping the eggs at 10 °C under a 12L12D photoperiod for more than 90 days. Post-diapause embryogenesis was resumed by incubating the eggs at 25 °C under a 16L8D photoperiod. In order to prevent hatching of fully developed embryos at high humidity, eggs containing embryos that developed pigmented eyespots and bristles were transferred to 43% relative humidity (RH) conditions [39], which were established using a saturated potassium carbonate solution [40]. For the experiments utilizing post-diapause stages, eggs retrieved from the field-collected twigs were also used.

### Eggshell observation using electron microscopy

For scanning electron microscopy (SEM), eggs were fixed with 4% paraformaldehyde solution containing 1% glutaraldehyde and were then treated with 1% osmium tetroxide. The samples were dehydrated using a series of ethanol solutions and finally treated with isoamyl acetate. After the samples were subjected to critical-point drying and platinum coating (10–15 nm), the eggshells were observed with an SEM apparatus (JEOL, JSM-6340F). For transmission electron microscopy (TEM), eggs were fixed and dehydrated as described above. Then, they were treated with propylene oxide and embedded in an epoxy resin (LUVEAK-812). Ultrathin sections (90 nm) were prepared with an ultramicrotome (Leica, Ultracut), stained with uranyl acetate and lead citrate, and observed with an H-7100 (Hitachi). For thickness measurement, we obtained transverse sections of eggshells 180–200 μm and 600–620 μm from the anterior tip. The precise position of these sections was obtained from the cumulative counter of the ultramicrotome. The thicknesses of the chorion and serosal cuticle were calculated from analyses of TEM images using Photoshop (6.0, Adobe).

### Nuclear staining

To observe the localization of the serosal cells, we performed whole-mount nuclear staining using the Feulgen reaction [41]. The eggs were fixed with Carnoy’s fixative at 60 °C for 30 min. After being washed with 70% ethanol, the eggs were hydrated and then subjected to hydrolysis in 1 N hydrochloric acid (HCl) at 60 °C for 30 min. Next, they were reacted with Schiff’s reagent for 30 min and rinsed with a solution of 0.5% sodium pyrosulfite in 50 mN HCl. The stained eggs were dehydrated using a series of ethanol solutions and finally transferred to methyl salicylate for microscopic observation.

### Measurement of eggshell permeability

As a measure of eggshell permeability, we assessed water-loss rates under dry conditions. Eggs at various developmental stages were transferred from the moistened chamber to a dry container adjusted to 43% RH using a saturated potassium carbonate solution [39]. Water-loss rates were calculated by measuring wet weights with an electronic microbalance (Mettler-Toledo, MT5) before and after the desiccation treatments; these quantities were expressed as weight loss per egg per day. In order to ensure accuracy in weighing, 10 eggs were weighed collectively.

### Measurement of eggshell cleavability

We constructed a force-gauge system to measure the cleavability of the eggshells (Fig. S1). Pressing eggs from outside generates turgor pressure, resulting in eggshell rupture and extrusion of the embryo. Eggs at various developmental stages were placed in the center of a small chamber (42 mm × 60 mm × 17 mm) and fixed to the bottom with double-sided adhesive tape. The center of the chamber lid had a hole (φ11 mm) to enable penetration of a pushing probe attached to a digital force gauge (Aikoh, RZ-1). The contact probe was made of resin and had a cylindrical body (φ10 mm) with a columnar tip (φ0.4 mm, Fig. S1). The probe was lowered vertically through the lid hole and gently pressed against the dorsal center of the egg. The load values that caused eggshell rupture were recorded. When the pushing force exceeded 1000 mN, the pressing was stopped because further pressing was predicted to cause the egg to burst rather than cleave along the hatching line.

### Statistics

All statistical analyses were performed using R (v 3.6.3) [42]. We performed statistical analyses based on a generalized linear model (GLM) framework. A Gaussian error distribution and an inverse-Gaussian error distribution were employed for eggshell thickness and water-loss rates, respectively. In order to assess the effects of developmental stages on eggshell cleavability, a nonparametric test for multiple comparisons, the Steel-Dwass test, was selected. In this test, eggs that did not rupture under 1000 mN pressure or that burst anywhere other than the hatching line were assumed to have a maximum value (1000 mN) and included in the tests.

## Results

### Eggshell of *C. facialis*

**Fig. 2:**
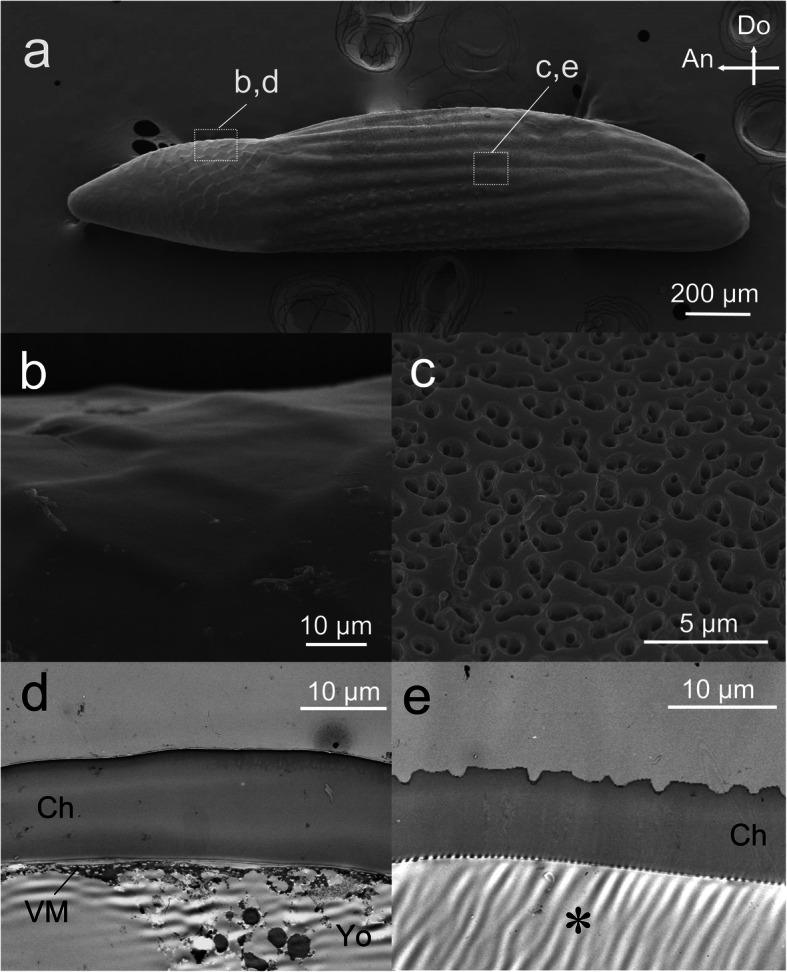
Fine structure of the eggshell on the oviposition date. **a** A whole SEM image of an eggshell. Arrows indicate the anterior (An) and dorsal (Do) directions. **b** A magnified SEM image of the dorsal middle region of the anterior part, which contains the future cleavage site. **c** An SEM image of the lateral region in the posterior part. **d** A TEM image of (**b**). **e** A TEM image of (**c**). In this sample, the egg contents have been removed (*). Abbreviations: Ch, chorion; VM, vitellin membrane: Yo, yolk

The eggshell of *C. facialis* was colorless and transparent, and the yolk granules and developing embryos were visible from the outside (Fig. 1b, c). As embryogenesis proceeded, the eggs became somewhat swollen at the posterior end by absorbing water (Fig. 1c), as reported in other cicada eggs [43]. At the time of hatching, the eggshell was always cleaved at a certain site that was located on the midline of the anterior one-fourth of the eggshell (Fig. 1c, orange broken line). Cleavage normally started from the dorsal apex and extended toward the ventral terminal via the tip region. SEM observation revealed that the anterior one-fourth of the eggshell has a distinct surface structure different from that of the posterior region (Fig. 2a). The anterior region was characterized by a smooth surface with polygonal shapes (Fig. 2b), while the posterior region was covered with numerous tiny holes in addition to horizontal wrinkles with small knobs (Fig. 2a, c). We did not find any distinct signs on the chorion surface at the future cleavage site (Fig. 2b). TEM observation of the transverse sections revealed that the thick chorion layer was composed of a homogeneously solid substance in both the anterior and posterior regions (Fig. 2d, e). Tiny holes found in the posterior part did not penetrate the chorion layer (Fig. 2e). In contrast to the chorion of many other insects [1, 2], no internal air spaces or cave-like structures were found. The transverse sections also verified that the eggshell had no obvious hatching line structure at the time of oviposition (Fig. 2d).

### Hatching line in the serosal cuticle

**Fig. 3:**
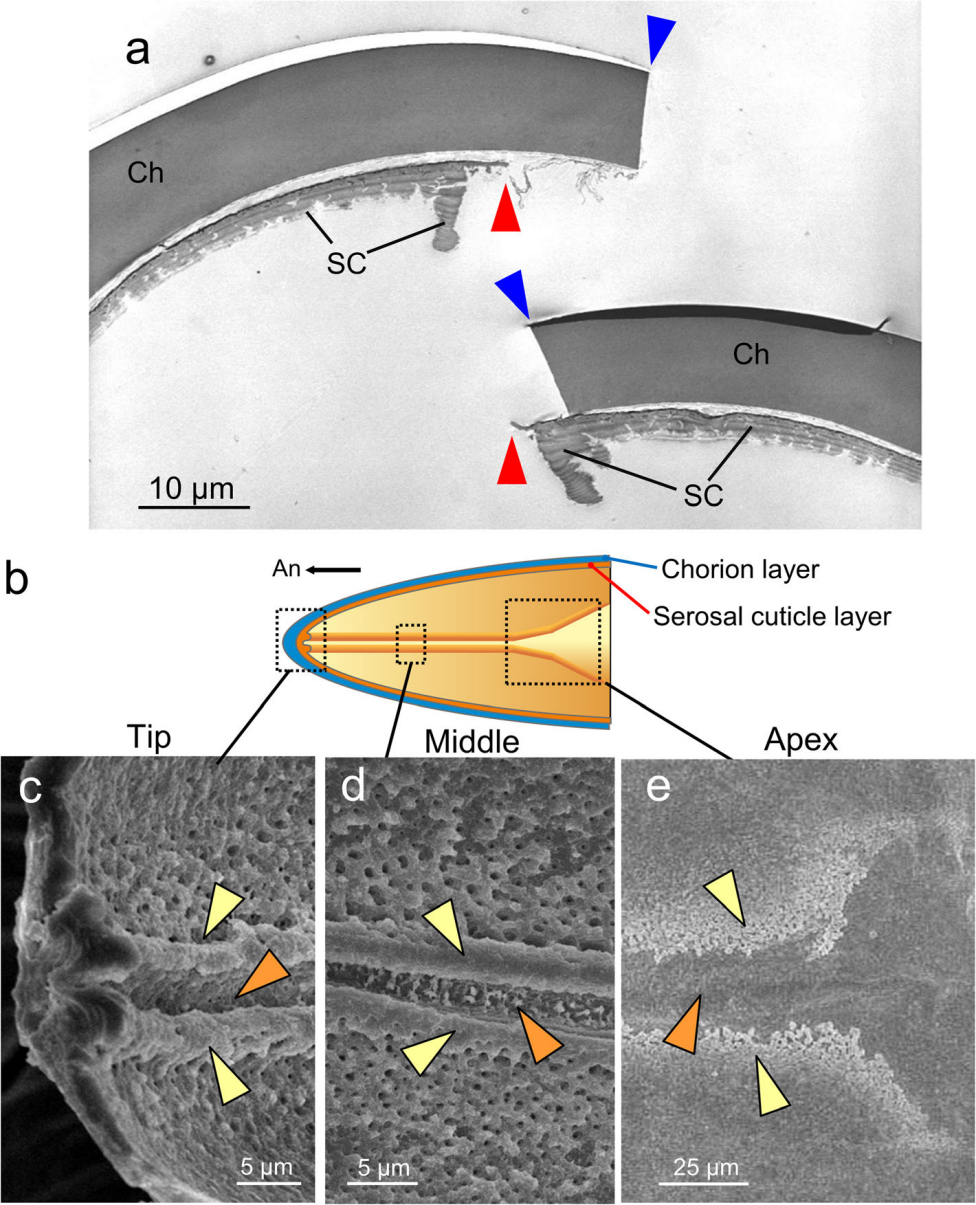
The hatching line in the serosal cuticle. **a** A TEM image of the anterodorsal cleavage site of a hatched eggshell. Blue and red arrowheads indicate the cleavage sites in the chorion and serosal cuticle, respectively. **b** A schematic representation of the inner surface structure of the anterodorsal part of the eggshell. **c-e** Interior SEM images of the eggshell of the fully developed eggs at the represented sites shown in (**b**). A furrow (orange arrowheads) flanked by two ridges (yellow arrowheads) runs along the midline of the anterior part. Abbreviations: Ch, chorion; SC, serosal cuticle

Next, we observed the fine structure of the cleavage site in hatched eggshells (Fig. 3a). The chorion was abruptly broken out without any visible modification. In this stage, however, beneath the chorion layer, there was an extraembryonic cuticular layer, namely, the serosal cuticle. The cleavage site in the serosal cuticle was remarkably thin and possessed protruding cuticular structures on both sides. This ridge-and-furrow structure extended along the midline of the anterior eggshell in accordance with the cleavage line (Fig. 3b-e). At the dorsal apex site, the structure became wider and shallower and gradually disappeared (Fig. 3e). These observations indicated that the ridge-and-furrow structure in the serosal cuticle defines the hatching line of *C. facialis*.

### Morphogenesis of the hatching line in the serosal cuticle

**Fig. 4:**
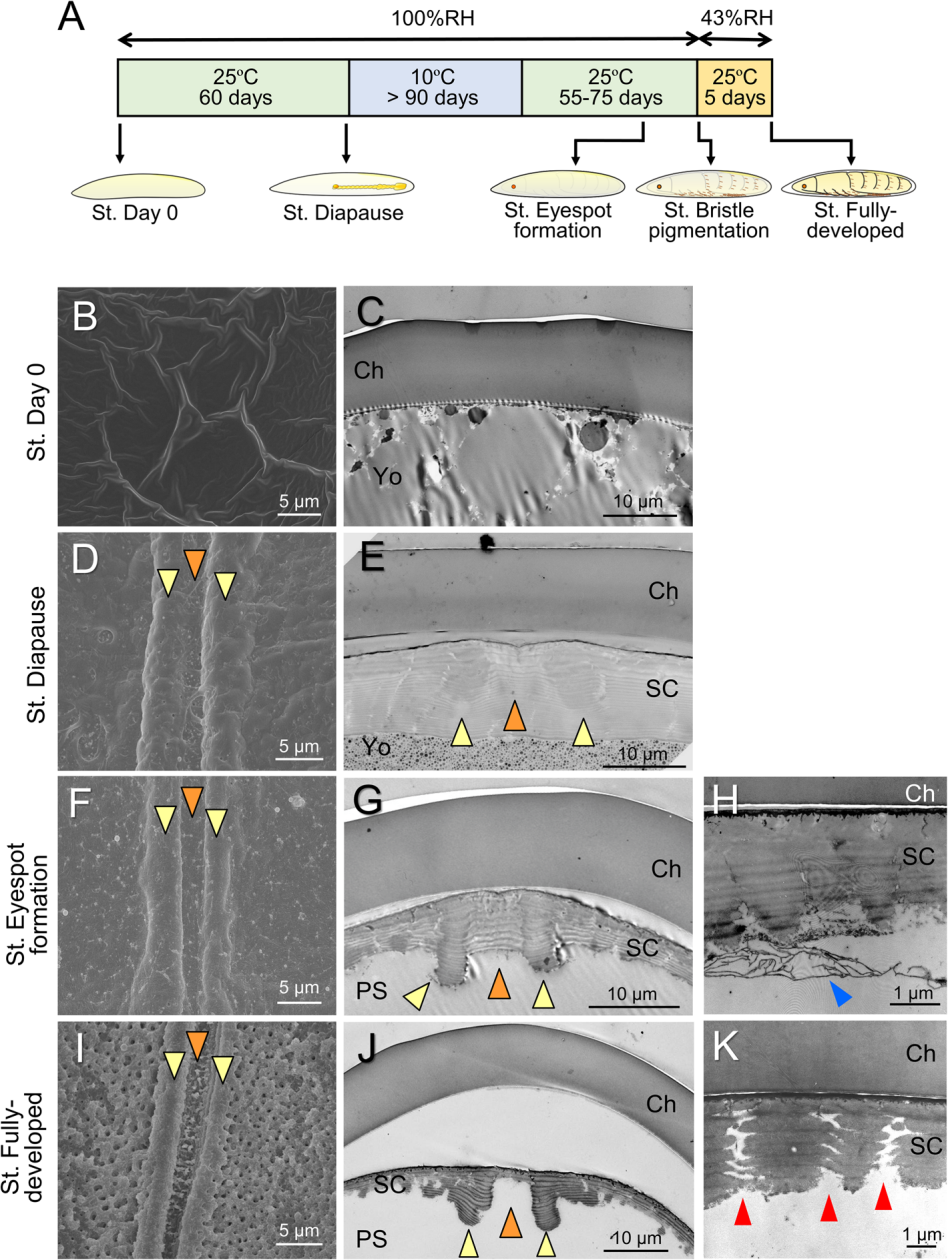
The developmental process of hatching-line formation in the serosal cuticle. **a** Time course of embryonic development and sampling points. **b, d, f, i** SEM images of the inner surface around the dorsal hatching line at each developmental stage. **c, e, g, j** Transverse TEM images of the area around the dorsal hatching line. Orange and yellow arrowheads indicate furrow and ridge positions, respectively. **h** A magnified image of the degrading serosal cuticle at the stage of eyespot formation (blue arrowhead). **k** A magnified image of pores that appeared in fully developed eggs (red arrowheads). Abbreviations: Ch, chorion; SC, serosal cuticle; PS, periembryonic space; Yo, yolk

**Fig. 5:**
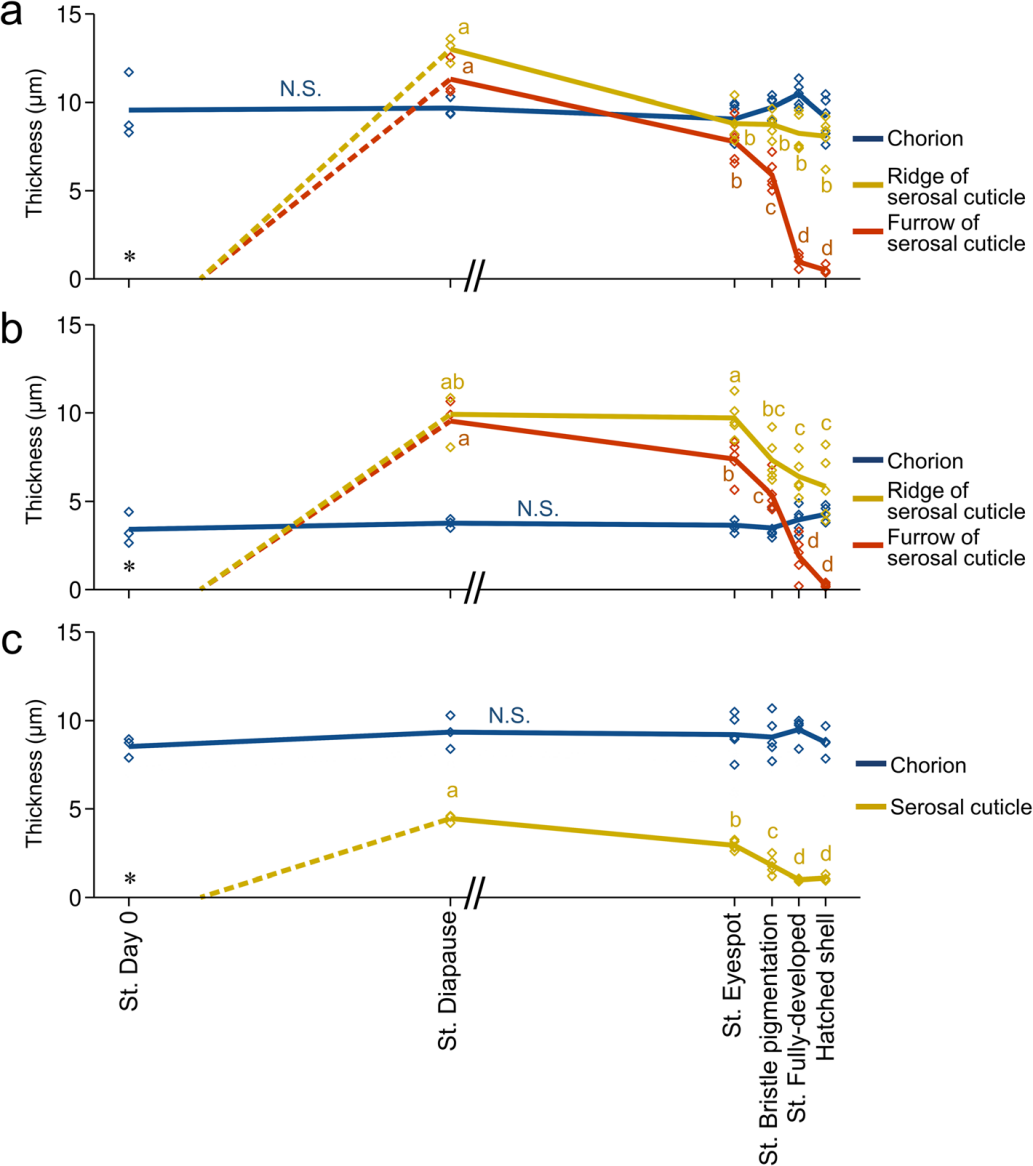
Developmental changes in eggshell thickness during embryogenesis. The thickness of the chorion and the serosal cuticle was measured on the dorsal hatching line 180–200 μm (**a**) or 600–620 μm (**b**) from the tip or at a lateral non-hatching-line site 180–200 μm from the tip (**c**). The horizontal axis indicates stages of embryonic development with a scale reflecting typical developmental time (see Fig. 4a), except that the period of low temperature needed for diapause termination is not included (//). Lines connect the mean values of each point in development, and diamonds show individual values (*N* = 3–5). No serosal cuticle was present on Day 0 (*). Although the cuticle layer became recognizable at approximately Day 15–20, we did not quantify the thickness change until the start of diapause (broken line). Different letters indicate statistically significant differences among developmental stages (likelihood ratio test of a generalized linear model, *P* < 0.05), while N.S. indicates no significance (*P* > 0.05)

To explore the process of formation of this peculiar structure, we surveyed eggshells across various embryonic stages, as shown in Fig. 4a. As seen in Fig. 2, no serosal cuticle was present on the day of oviposition (Fig. 4b, c). The deposition of the serosal cuticle was identifiable approximately 15–20 days after oviposition. By the diapause stage, in which embryonic development is arrested at the early germ-band stage [34], a thick, lamellar serosal cuticle had formed beneath the chorion layer (Fig. 4d, e). Interestingly, the future cleavage site (furrow) was covered with a thick cuticle layer, and the future ridge positions slightly bulged. During post-diapause development, the inside of the serosal cuticle layer was partially scraped away, and the furrow-like structure appeared (Fig. 4f, h). As the completion of embryonic development approached, the cuticle layer was further reduced, especially in the furrow position, whereas the ridge positions persisted, and as a result, distinct ridge and furrow structures were shaped (Fig. 4i, j). In this stage, many pores that penetrated the serosal cuticle were observed, especially around the hatching line (Fig. 4k). The developmental changes in the thickness of the chorion and serosal cuticle were quantified at the middle dorsal site (Fig. 5a) and the dorsal apex site (Fig. 5b) within the hatching line and at a lateral site away from the hatching line (Fig. 5c). The thickness of the chorion in these three positions did not change across developmental stages. The serosal cuticle reached its maximum thickness at the diapause stage. The relatively thick cuticle layer persisted until approximately the time of eyespot formation, but thereafter, the rate of cuticle degradation was accelerated toward the end of embryogenesis, especially in the furrow position. At the time of hatching, the serosal cuticle at the furrow position was reduced to less than 5% of maximum thickness, whereas the ridge positions retained approximately 60% of maximum thickness. Degradation of the serosal cuticle took place throughout the eggshell and was not restricted to the hatching line (Fig. 5c).

**Fig. 6:**
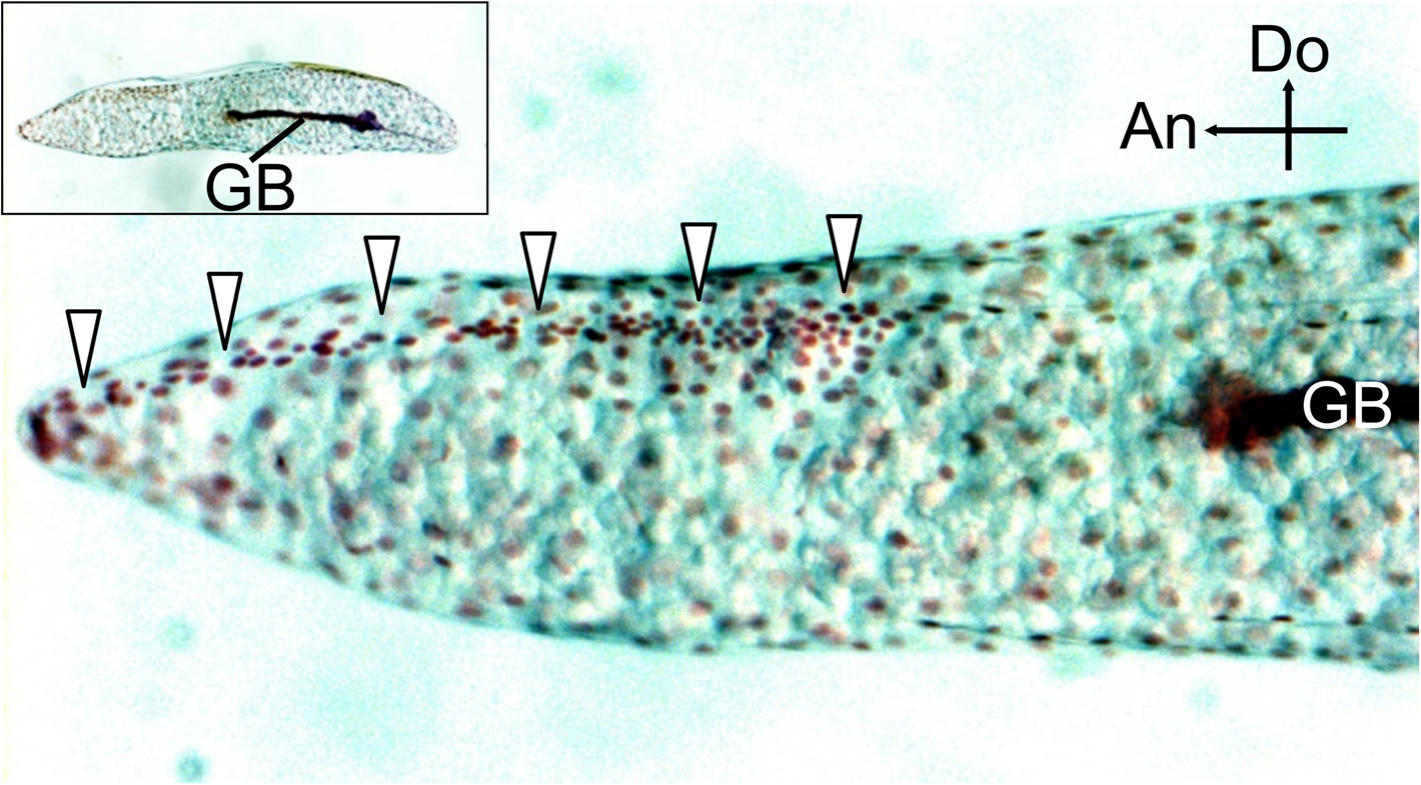
Prior casting of the hatching line by serosal cells during early embryogenesis. Nuclei in the germ-band (GB) elongation stage (20 days after oviposition) were visualized by Feulgen staining. An image of the whole egg is displayed in the inset. The nuclei of serosal cells were distinctly gathered along the developing hatching line (arrowheads). Crossed arrows indicate the anterior (An) and dorsal (Do) directions

These results demonstrated that the structure of the serosal cuticle dynamically changes during embryogenesis and that the hatching line on the serosal cuticle is shaped by cutting from a thick cuticle layer rather than by casting ab initio. A base for the ridge-and-furrow structure was embedded in advance during the formation of the thick cuticle. In the serosal cuticle layer at the diapause stage, the outlines of the developing ridge-and-furrow structure were distinguishable as slightly electron-dense regions (Fig. 4e, orange and yellow arrowheads). In addition, at the stage of serosal cuticle deposition (20 days after oviposition), the serosal cells were densely gathered along the future hatching line (Fig. 6), suggesting that they were preparing special architectures in this stage.

### Functional change during the formation of the hatching line

**Fig. 7:**
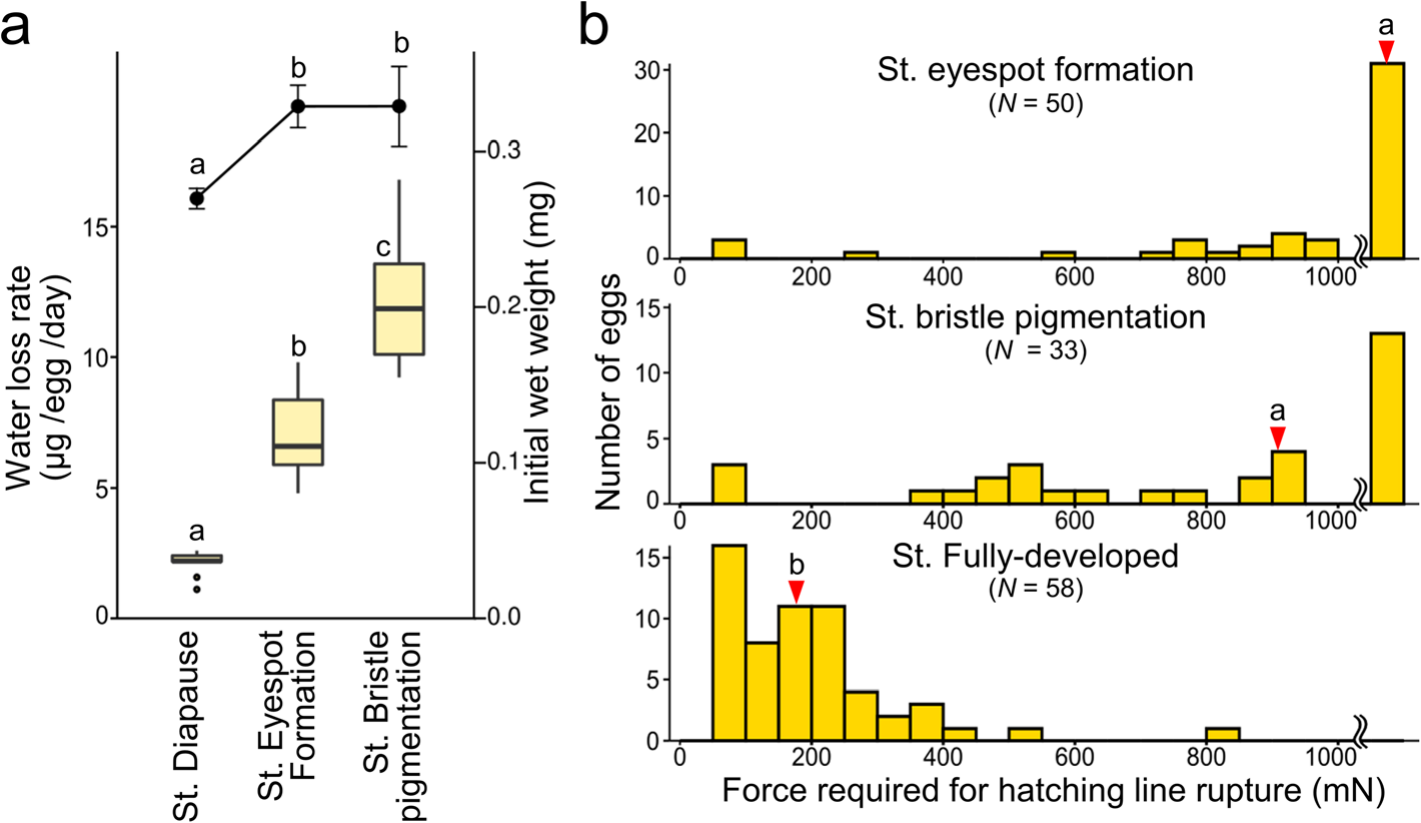
Dynamic changes in eggshell properties with serosal cuticle degradation. **a** Change in water-loss rates. Box plots indicate water-loss rates after 5 days of exposure to 43% relative humidity conditions; these rates were assessed gravimetrically in eggs at various developmental stages. The initial weights at each developmental stage are also shown by line plots. Different letters indicate statistically significant differences (*N* = 12 samples (each containing 10 eggs), likelihood ratio test of a generalized linear model, *P* < 0.05). **b** Change in hatching-line cleavability. The pushing force needed to cause a rupture of the hatching line was measured. The number of eggs that were not ruptured by less than 1000 mN and the number that burst in locations other than the hatching line are shown in the rightmost bars. Median values for each developmental stage are indicated by red triangles, and different letters indicate significant differences (Steel-Dwass test, *P* < 0.05)

Do these dynamic morphological changes in the serosal cuticle lead to functional consequences? As an index of eggshell permeability, we assessed the changes in water-loss rates with serosal cuticle degradation. Weight-loss rates during a 5-day desiccation period at 43% RH were compared among eggs in the diapause, eyespot formation, and bristle pigmentation stages. It should be noted that eggs in the diapause stage had heavier initial weights because the eggs absorbed water during the early stages of the post-diapause development (Fig. 7a). Water-loss rates in the diapause stage, in which the serosal cuticle reached maximum thickness, stayed under 2.2 μg/day (comparable to 0.8% of total egg weight/day). As embryogenesis proceeded, water-loss rates drastically increased in tandem with cuticle degradation (see Fig. 5), and the desiccation treatment caused 7.0 μg (2.1%) and 12.1 μg (3.7%) loss/day in the eyespot formation stage and the bristle pigmentation stage, respectively.

Next, we investigated the changes in the cleavability of the eggshell. In this experiment, we measured the pushing force necessary to generate sufficient turgor pressure for the hatching line to rupture (see Methods and Fig. S1 for details). In the eyespot formation stage, the hatching line was rarely cleaved by less than 1000 mN of force on the egg (Fig. 7b). At the bristle pigmentation stage, the majority of eggshells were cleaved at the hatching line when compressed, but the necessary amount of force was still high. In the fully developed eggs with complete ridge-and-furrow structures, the hatching line became fragile enough to be cleaved by a force of less than 500 mN. These results demonstrated that eggshell properties such as robustness and permeability dynamically changed in association with hatching-line formation accompanying serosal cuticle degradation.

## Discussion

### The hatching line in the serosal cuticle

To facilitate the safe hatching of neonates, many insect eggshells have special structures, known as hatching lines and opercula, that create weakness at the site to be separated [1, 2]. These structures are usually part of the maternally derived chorion layer and are therefore carried throughout the egg period. In the present study, we found that cicada eggs had a hatching line in the serosal cuticle, whereas no obvious weakness-causing structures were found in the chorion. The serosal cuticle, a membrane made of chitin and proteins, is deposited beneath the chorion by the extraembryonic serosa during embryonic development [23, 24, 44, 45]. This secondary cuticle layer is commonly observed in most insect taxa and is considered to augment or replace the protective functions of the chorion. Until now, the construction of hatching lines in the serosal cuticle was reported only in a carabid beetle, *Carabus insulicola* [46]. In that beetle, picric acid–stainable lines in the serosal cuticle were found to become the rupture site, but the fine structure and developmental process of this hatching line have not been investigated. Here, we found that the hatching line of cicada eggs is composed of a fine furrow accompanied by two ridges, one on each side (Fig. 3). We also demonstrated that the ridge-and-furrow pattern is excavated from an initially thick lamellar cuticle rather than being piled up on a thin base layer (Figs. 4, 5). The degradation of the serosal cuticle occurred to some extent during the middle to late phase of embryonic development after diapause termination. However, substantial excavation of the hatching line occurred in a relatively short time at the terminal phase of embryogenesis (Fig. 5).

Degradation of the serosal cuticle toward the completion of embryogenesis has been explicitly described in some orthopterans [12, 47] and coleopterans [48]. Chitin-degrading enzymes, known as hatching enzymes, are believed to be secreted from the pleuropodia [12, 49, 50] or serosa [51, 52]. The pleuropodia of a periodical cicada, *Magicicada cassini*, develop in mid-embryonic stages around the time of katatrepsis and degenerate in the following embryonic stages [53]. This suggests that the pleuropodia are unlikely to be the main structures involved in serosal cuticle degradation in cicadas. It has been reported that hatching enzymes affect the inner but not the outer layers of the multilayered serosal cuticle, which have distinctly different properties of color and stainability [12, 48]. Although all layers of the serosal cuticle of *C. facialis* eggs are colorless and transparent, we found that the developing furrow-and-ridge structure embedded in the thick lamellar cuticle is distinguishable by TEM observation at the time of cuticle deposition. This implies that the hatching line is made of qualitatively different materials resistant to digestion by hatching enzymes.

Although the exact roles of the furrow-and-ridge shape of the hatching line are not clear at present, we speculate about its contribution to hatching. First, we could not find any sign of weakness in the chorion layer throughout the egg period. Nevertheless, the chorion always ruptures along the hatching line of the serosal cuticle at hatching. We observed that, except during the terminal phase of embryogenesis, pressing the egg with a strong force caused the eggshell to rupture in locations other than the hatching line, even before serosal cuticle formation. This implies that the chorion around the hatching line also becomes fragile toward the completion of embryogenesis. Therefore, the furrow part of the hatching line serves as a site of weakness itself and may also affect the chorion by secreting unknown effectors. In fact, the serosal cuticle around the hatching line was observed to possess numerous fine canals (Fig. 4i-k), which might potentially serve as a route of secretion. On the other hand, the ridge structure may support the ability of nymphs to cleave the eggshell. At the time of hatching, pharate nymphs that bear an embryonic cuticle, called pronymphs, start forward movement by vermicular motion, but they lack any apparent structure with which to open the eggshell, for example, a structure resembling an egg burster or egg tooth [13, 20, 54]. The two ridges of the serosal cuticle are presumed to receive the force applied by the movement of a nymph and effectively focus it on the cleavage line; otherwise, the force might disperse across the eggshell.

### The hatching line in the serosal cuticle allows dynamic changes in eggshell properties

The complex process of hatching-line formation in *C. facialis* may provide an advantage in changing the properties of the eggshell during embryogenesis. Modulation of eggshell functions by production and degradation of the serosal cuticle has been demonstrated in several insect species [24]. The underlying serosal cuticle has been shown to reduce the water and oxygen penetrability of eggshells in coleopteran and lepidopteran species [16, 55, 56]. Desiccation tolerance in mosquito eggs is attained concomitantly with serosal cuticle deposition [25–27]. Moreover, a clear contribution of the serosal cuticle to desiccation tolerance was experimentally demonstrated in the red flour beetle, *Tribolium castaneum*, by RNAi-based knockdown of genes involved in serosal cuticle synthesis [52, 57]. In *C. facialis* eggs, deposition of the serosal cuticle could be observed after germ-band invagination (Fig. 6), as is typical in hemimetabolous insects [58–60], and the thick cuticle layer eventually formed by the time the egg reached the diapause stage. We verified that water-loss rates, as a measure of eggshell permeability, remained at remarkably low levels in this stage compared to the later stages, when the cuticle was degraded (Fig. 7a). Because of low metabolic demand during diapause, the production of thick, impermeable eggshells is likely to be an adaptation against water loss and penetration of pathogens during prolonged developmental periods at the expense of oxygen supply. This idea is supported by the finding that diapause-destined eggs have eggshells with lower permeability to oxygen and water than non-diapause-destined eggs in grasshoppers [6, 61] and silkworms [16].

In the late phase of embryogenesis, the oxygen requirement increases to support development and neonate activity [5], and a tough eggshell can be an obstacle to hatching [12]. The degradation of the serosal cuticle and the excavation of the furrow-and-ridge structure in *C. facialis* appear to fulfill these demands. The permeability of the eggshell was shown to increase toward hatching (Fig. 7a). We also demonstrated that eggshells underwent a physical change to facilitate rupture at the hatching line (Fig. 7b). Taken together, our findings suggest that the formation of the hatching line in the serosal cuticle enables the eggshell to change its properties flexibly in order to reconcile conflicting demands during embryogenesis, i.e., to be impermeable during early to mid-embryogenesis, including diapause, and permeable or fragile in the late embryonic phase.

### Evolution of the hatching line in the serosal cuticle of cicadas

One question that our findings raise is why this unique structure has evolved in cicadas. In carabid eggs, the other known example, the chorion is sometimes peeled off and replaced with the serosal cuticle [46]. Therefore, the situation would not be the same as in the cicada eggs, in which the robust chorion persists until hatching. The primary reason in cicadas may be related to the extremely long egg period. The egg period of temperate cicadas that overwinter in embryonic diapause typically ranges from 9 to 12 months, while a period of 1–3 months is still needed even in cicada species without embryonic diapause [31, 35]. Our previous study addressing *C. facialis* embryonic development revealed that its prolonged egg duration is attributable not only to a long overwintering diapause but also to an extraordinarily slow rate of embryonic development [34]. Therefore, the oxygen demand during the non-diapause period is predicted to be low compared to that of other insects, although it would decrease further in the diapause period, as mentioned above. If a hatching line is formed in the chorion, although its area is limited, it can be a nonnegligible cause of water loss or pathogen intrusion for eggs with extremely long egg periods. We found that cicada eggs do not bear chorionic structures specialized for gas exchange, such as aeropyles and air cavities, which are common to a diverse range of insects [1, 2, 20]. This suggests that cicadas favor eggshells serving as a robust barrier at the expense of gas exchange. On the other hand, some cicada species, including *C. facialis*, need to rapidly emerge from the eggshell in response to rain to ensure that the nymphs can burrow into wet, soft ground [36–38]. These opposite needs at hatching versus earlier stages may be fulfilled with a robust shell by locally varying the destructibility of the serosal cuticle, rather than the chorion, to form a hatching line.

In this study, we focused on the eggshell of *C. facialis*, which undergoes embryonic diapause and humidity-inducible hatching. In contrast to *C. facialis*, some temperate cicada species use a strategy of hatching within the oviposition year, without overwintering in diapause [31, 53, 62, 63]. In addition, although *C. facialis* lays eggs in dead twigs [33], some other cicadas choose live plant tissues for oviposition [64, 65]. Their eggs appear to encounter various moist environments and are unlikely to hatch in response to humidity cues, implying that they might face different types of adaptive demands than *C. facialis*. Such variation in oviposition site selection is known even within the genus *Cryptotympana* [64, 66]. Therefore, to clarify the adaptive significance and evolutionary origin of hatching lines in the serosal cuticle, comparative studies must examine these cicada species with various diapause and hatching traits and address other Auchenorrhyncha species.

## Conclusions

This study demonstrated that the development of a peculiar structure in the serosal cuticle of *Cryptotympana facialis* enables flexible modification of eggshell properties during the long embryonic period. These findings reveal a novel mode of environmental adaptation through sophisticated insect eggshells.

## Supplementary Information

**Additional file 1: Fig. S1.** A schematic illustration of the system to measure hatching linebreaking forces.

## Data Availability

The datasets used and/or analyzed during the current study are available from the corresponding author on reasonable request.

## References

[1] Hinton HE (1981). Biology of insect eggs.

[2] Margaritis LH, Kerkut GA, Gilbert LI (1985). Structure and physiology of the eggshell. Comprehensive insect physiology biochemistry and pharmacology.

[3] Zeh DW, Zeh JA, Smith RL (1989). Ovipositors, amnions and eggshell architecture in the diversification of terrestrial arthropods. Q Rev Biol.

[4] Hinton HE (1969). Respiratory systems of insect egg shells. Annu Rev Entomol.

[5] Woods HA (2010). Water loss and gas exchange by eggs of *Manduca sexta*: trading off costs and benefits. J Insect Physiol.

[6] Browning TO (1969). Permeability to water of the shell of the egg of *Locusta migratoria migratorioides*, with observations on the egg of *Teleogryllus commodus*. J Exp Biol.

[7] Browning TO (1969). The permeability of the shell of the egg of *Teleogryllus commodus* measured with the aid of tritiated water. J Exp Biol.

[8] Yoder JA, Denlinger DL (1992). Water vapour uptake by diapausing eggs of a tropical walking stick. Physiol Entomol.

[9] Niikawa K, Takeda M (1996). Water absorption by diapause and nondiapause eggs in two *Velanfictorus* species (Orthoptera: Gryllidae). Appl Entomol Zool.

[10] Rotheram S (1973). The surface of the egg of a parasitic insect. I. The surface of the egg and first instar larva of *Nemeritis*. Proc R Soc London - Biol Sci.

[11] Buckner JS, Freeman TP, Ruud RL, Chu CC, Henneberry TJ (2002). Characterization and functions of the whitefly egg pedicel. Arch Insect Biochem Physiol.

[12] Slifer EH (1937). The origin and fate of the membranes surrounding the grasshopper egg; together with some experiments on the source of the hatching enzyme. J Cell Sci.

[13] Pérez-de la Fuente R, Engel MS, Azar D, Peñalver E (2019). The hatching mechanism of 130-million-year-old insects: an association of neonates, egg shells and egg bursters in *Lebanese amber*. Palaeontology.

[14] Woods HA, Singer MS (2001). Contrasting responses to desiccation and starvation by eggs and neonates of two Lepidoptera. Physiol Biochem Zool.

[15] Jagadeeshan S, Singh RS (2007). Rapid evolution of outer egg membrane proteins in the *Drosophila melanogaster* subgroup: a case of ecologically driven evolution of female reproductive traits. Mol Biol Evol.

[16] Sonobe H, Matsumoto A, Fukuzaki Y, Fujiwara S (1979). Carbohydrate metabolism and restricted oxygen supply in the eggs of the silkworm, *Bombyx mori*. J Insect Physiol.

[17] Kim SE (1987). Changes in eggshell permeability to oxygen during the early developmental stages in diapause eggs of *Bombyx mori*. J Insect Physiol.

[18] Zrubek B, Woods HA (2006). Insect eggs exert rapid control over an oxygen-water tradeoff. Proc R Soc B Biol Sci.

[19] Margaritis LH, Kafatos FC, Petri WH (1980). The eggshell of *Drosophila melanogaster*. Fine structure of the layers and regions of the wild-type eggshell. J Cell Sci.

[20] Cobben RH (1968). Evolutionary trends in Heteroptera part I: eggs, architecture of the shell, gross embryology and eclosion.

[21] Matesco VC, Fürstenau BBRJ, Bernardes JLC (2009). Morphological features of the eggs of Pentatomidae (Hemiptera: Heteroptera). Zootaxa..

[22] Fukui M, Fujita M, Tomizuka S, Mashimo Y, Shimizu S, Lee CY, Murakami Y, Machida R (2018). Egg structure and outline of embryonic development of the basal mantodean, *Metallyticus splendidus* Westwood, 1835 (Insecta, Mantodea, Metallyticidae). Arthropod Struct Dev..

[23] Machida R (2006). Evidence from embryology for reconstructing the relationships of hexapod basal clades. Arthropod Syst Phylogeny.

[24] Panfilio KA (2008). Extraembryonic development in insects and the acrobatics of blastokinesis. Dev Biol.

[25] Rezende GL, Martins AJ, Gentile C, Farnesi LC, Pelajo-Machado M, Peixoto AA, Valle D (2008). Embryonic desiccation resistance in *Aedes aegypti*: presumptive role of the chitinized serosal cuticle. BMC Dev Biol.

[26] Goltsev Y, Rezende GL, Vranizan K, Lanzaro G, Valle D, Levine M (2009). Developmental and evolutionary basis for drought tolerance of the *Anopheles gambiae* embryo. Dev Biol.

[27] Vargas HCM, Farnesi LC, Martins AJ, Valle D, Rezende GL (2014). Serosal cuticle formation and distinct degrees of desiccation resistance in embryos of the mosquito vectors *Aedes aegypti*, *Anopheles aquasalis* and *Culex quinquefasciatus*. J Insect Physiol.

[28] Slifer EH (1938). The formation and structure of a special water absorbing area in the membranes covering the grasshopper egg. J Cell Sci.

[29] Madhavan MM (1974). Structure and function of the hydropyle of the egg of the bug, *Sphaerodema molestum*. J Insect Physiol.

[30] Mtow S, Machida R (2018). Development and ultrastructure of the thickened serosa and serosal cuticle formed beneath the embryo in the stonefly *Scopura montana* Maruyama, 1987 (Insecta, Plecoptera, Scopuridae). Arthropod Struct Dev.

[31] Beamer R (1928). Studies on the biology of Kansas Cicadidae. Univ Kansas Sci Bull.

[32] Myers JG (1929). Insect singers: a natural history of the cicadas.

[33] Moriyama M, Matsuno T, Numata H (2016). Dead-twig discrimination for oviposition in a cicada, *Cryptotympana facialis* (Hemiptera: Cicadidae). Appl Entomol Zool.

[34] Moriyama M, Numata H (2008). Diapause and prolonged development in the embryo and their ecological significance in two cicadas, *Cryptotympana facialis* and *Graptopsaltria nigrofuscata*. J Insect Physiol.

[35] Moriyama M, Numata H (2019). Ecophysiological responses to climate change in cicadas. Physiol Entomol.

[36] Moriyama M, Numata H (2006). Induction of egg hatching by high humidity in the cicada *Cryptotympana facialis*. J Insect Physiol.

[37] Moriyama M, Numata H (2011). A cicada that ensures its fitness during climate warming by synchronizing its hatching time with the rainy season. Zool Sci.

[38] Moriyama M, Numata H (2015). Urban soil compaction reduces cicada diversity. Zool Lett.

[39] Moriyama M, Numata H (2010). Desiccation tolerance in fully developed embryos of two cicadas, *Cryptotympana facialis* and *Graptopsaltria nigrofuscata*. Entomol Sci.

[40] Winston PW, Bates DH (1960). Saturated solutions for the control of humidity in biological research. Ecology..

[41] Lyon HO, Schulte EK, Prento P, Barer MR, Béné MC (2002). Standardized staining methods: Feulgen-Rossenbeck reaction for desoxyribonucleic acid and periodic acid-Schiff (PAS) procedure. Biotech Histochem.

[42] R Core Team. R: A language and environment for statistical computing. R Foundation for Statistical Computing, Vienna, Austria. https://www.R-project.org/. Accessed 15 Feb 2021.

[43] White J, Lloyd M (1981). On the stainability and mortality of periodical cicada eggs. Am Midl Nat.

[44] Machida R, Ikeda Y, Tojo K (2002). Evolutionary changes in developmental potentials of the embryo proper and embryonic membranes in hexapoda: a synthesis revised. Proc Arthropod Embryol Soc Japan.

[45] Schmidt-Ott U, Kwan CW (2016). Morphogenetic functions of extraembryonic membranes in insects. Curr Opin Insect Sci.

[46] Kobayashi Y, Niikura K, Oosawa Y, Takami Y (2013). Embryonic development of *Carabus insulicola* (Insecta, Coleoptera, Carabidae) with special reference to external morphology and tangible evidence for the subcoxal theory. J Morphol.

[47] Furneaux PJ, James CR, Potter S (1969). The egg shell of the house cricket (*Acheta domesticus*): an electronmicroscope study. J Cell Sci.

[48] Kobayashi Y (2003). Development of the pleuropodia in the embryo of the glowworm *Rhagophthalmus ohbai* (Rhagophthalmidae, Coleoptera, Insecta), with comments on their probable function. Proc Arthropod Embryol Soc Japan..

[49] Slifer EH (1938). A cytological study of the pleuropodia of *Melanoplus differentialis* (Orthoptera, Acrididae) which furnishes new evidence that they produce the hatching enzyme. J Morphol.

[50] Konopová B, Buchberger E, Crisp A (2020). Transcriptome of pleuropodia from locust embryos supports that these organs produce enzymes enabling the larva to hatch. Front Zool.

[51] Zhu Q, Arakane Y, Beeman RW, Kramer KJ, Muthukrishnan S (2008). Functional specialization among insect chitinase family genes revealed by RNA interference. Proc Natl Acad Sci U S A.

[52] Jacobs CGC, Braak N, Lamers GEM, van der Zee M (2015). Elucidation of the serosal cuticle machinery in the beetle *Tribolium* by RNA sequencing and functional analysis of *Knickkopf1*, *Retroactive* and *Laccase2*. Insect Biochem Mol Biol.

[53] Strauß J, Lakes-Harlan R (2006). Embryonic development of pleuropodia of the cicada, *Magicicada cassini*. J Insect Sci.

[54] Rakitov R (2018). Pronymphs, hatching, and proboscis assembly in leafhoppers and froghoppers (Hemiptera, Cicadellidae and Aphrophoridae). Arthropod Struct Dev..

[55] Lincoln DCR (1961). The oxygen and water requirements of the egg of *Ocypus olens* Müller (Staphylinidae, Coleoptera). J Insect Physiol.

[56] Woods HA, Bonnecaze RT, Zrubek B (2005). Oxygen and water flux across eggshells of *Manduca sexta*. J Exp Biol.

[57] Jacobs CGC, Rezende GL, Lamers GEM, van der Zee M (2013). The extraembryonic serosa protects the insect egg against desiccation. Proc Biol Sci.

[58] Tojo K, Machida R (1997). Embryogenesis of the mayfly *Ephemera japonica* McLachlan (Insecta: Ephemeroptera, Ephemeridae), with special reference to abdominal formation. J Morphol.

[59] Masumoto M, Machida R (2006). Development of embryonic membranes in the silverfish *Lepisma saccharina* Linnaeus (Insecta: Zygentoma, Lepismatidae). Tissue Cell.

[60] Suzuki K, Watanabe Y, Tojo K (2020). Embryogenesis of the damselfly *Euphaea yayeyamana* Oguma (Insecta: Odonata: Euphaeidae), with special reference to the formation of their larval abdominal “gill-like” appendages. Entomol Sci..

[61] Gehrken U, Doumbia YO (1996). Diapause and quiescence in eggs of a tropical grasshopper *Oedaleus senegalensis* (Krauss). J Insect Physiol.

[62] Azuma S (1976). Biological studies of the sugar cane cicada, *Mogannia minuta* Matsumura, with special reference to its occurrence in relation to changes of commercial sugar cane varieties in Okinawa. Sci Bull Fac Agric Univ Ryukyus.

[63] Williams KS, Simon C (1995). The ecology, behavior, and evolution of periodical cicadas. Annu Rev Entomol.

[64] Hou Z, Zhong H, Nansen C, Wei C (2019). An integrated analysis of hyperspectral and morphological data of cicada ovipositors revealed unexplored links to specific oviposition hosts. Zoomorphology..

[65] Clay K, Shelton AL, Winkle C (2009). Differential susceptibility of tree species to oviposition by periodical cicadas. Ecol Entomol.

[66] Hayashi M, Saisho Y (2011). The Cicadidae of Japan.

