# Supplementary figures and images for: The formation of a hatching line in the serosal cuticle confers multifaceted adaptive functions on the eggshell of a cicada
Source: Zoological Lett. 2021 May 13;7:8. doi: 10.1186/s40851-021-00178-8 (PMC8117633; doi:10.1186/s40851-021-00178-8)

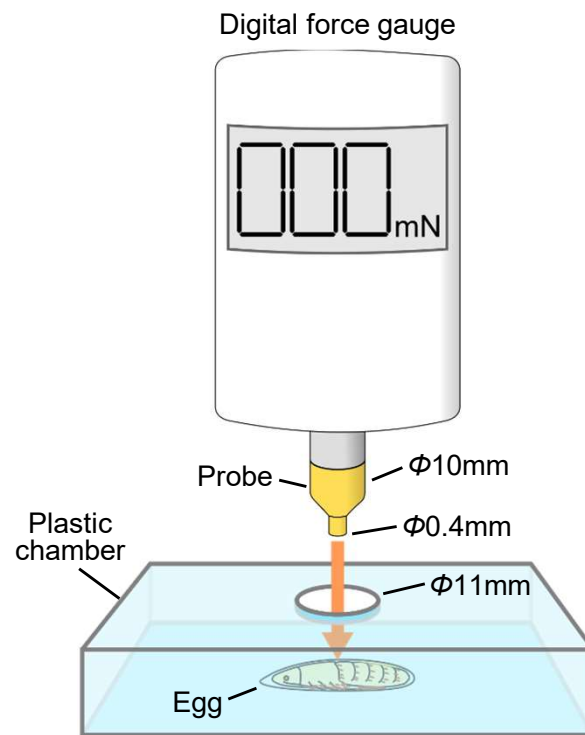

**Fig. S1** A schematic illustration of the system to measure hatching line-breaking forces.

Supplement: Supplementary file 1 — Additional file 1: Fig. S1. A schematic illustration of the system to measure hatching linebreaking forces. [file 40851_2021_178_MOESM1_ESM.pdf]
